# Photography by Cameras Integrated in Smartphones as a Tool for Analytical Chemistry Represented by an Butyrylcholinesterase Activity Assay

**DOI:** 10.3390/s150613752

**Published:** 2015-06-11

**Authors:** Miroslav Pohanka

**Affiliations:** Faculty of Military Health Sciences, University of Defense, Trebesska 1575, Hradec Kralove CZ-50001, Czech Republic; E-Mail: miroslav.pohanka@unob.cz or miroslav.pohanka@gmail.com; Tel.: +420-973-253-091

**Keywords:** photography, imagination, colorimetry, digital photography, mobile phone, RGB, CMYK, photometry, diagnosis, acetylcholinesterase, butyrylcholinesterase, naked eye detection

## Abstract

Smartphones are popular devices frequently equipped with sensitive sensors and great computational ability. Despite the widespread availability of smartphones, practical uses in analytical chemistry are limited, though some papers have proposed promising applications. In the present paper, a smartphone is used as a tool for the determination of cholinesterasemia *i.e.*, the determination of a biochemical marker butyrylcholinesterase (BChE). The work should demonstrate suitability of a smartphone-integrated camera for analytical purposes. Paper strips soaked with indoxylacetate were used for the determination of BChE activity, while the standard Ellman’s assay was used as a reference measurement. In the smartphone-based assay, BChE converted indoxylacetate to indigo blue and coloration was photographed using the phone’s integrated camera. A RGB color model was analyzed and color values for the individual color channels were determined. The assay was verified using plasma samples and samples containing pure BChE, and validated using Ellmans’s assay. The smartphone assay was proved to be reliable and applicable for routine diagnoses where BChE serves as a marker (liver function tests; some poisonings, *etc.*). It can be concluded that the assay is expected to be of practical applicability because of the results’ relevance.

## 1. Introduction

Colorimetric techniques are suitable for the determination of various substrates and they can be performed in combination with a naked eye examination or with some device measuring spectral properties. Disparate analytes can be determined using colorimetry. For example, arsenic (3+) in water samples [1], cobalt (2+) in water samples [2] and nutrients in corn [3] can be assayed by colorimetry. Compared to other spectroscopic techniques, colorimetry provides more complex but not easy to process data [4]. When a Red–Green–Blue (RGB) color model is used independent values of the color channels can be obtained for the assay. Other color models such as Cyan–Magnetta–Yellow–Key/black (CMYK) are used for specific purposes such as color printing, but RGB is probably the most common one used in digital photography.

Two cholinesterases are currently known: acetylcholinesterase (AChE; EC 3.1.1.7) and butyrylcholinesterase (BChE; EC 3.1.1.8). AChE is involved in the termination of cholinergic neurotransmission by hydrolysis of the transmitter acetylcholine [5]. Compared to AChE, BChE has no clearly visible function in the body, except of detoxification of some drugs and secondary metabolites from plants [6,7]. Both cholinesterases can be used as a marker for poisoning by nerve agents such as sarin or VX and some pesticides such as malaoxon or carbofuran [8,9]. Plasmatic levels of BChE, cholinesterasemia, can be used as a liver function test besides for the diagnosis of poisoning [10]. Determination of BChE activity in the presence of dibucaine or fluoride serves as a test for estimation of sensitivity to myorelaxants such as succinylcholine or mivacurium [11,12].

In this paper, the suitability of photography by a camera integrated in a smartphone as a tool for analytical chemistry is considered. The use of the camera integrated in a smartphone for a BChE activity assay is an original idea, even though some proposals for the use of smartphones were described in recent times. Chemical sensors [13], water quality sensors [14], immunosensors [15] and blood analysis sensors [16] can be mentioned as devices being paired with smartphones. The use of Google Glass for chlorophyll measurement is another interesting adaptation of camera-containing devices [17]. In this work, BChE activity in plasma is taken as a biochemical marker that can be determined out of the lab just using a smartphone. The assay works as an applied colorimetry assay with simplification of the output value. The proposal is aimed at the fact that smartphones or any other similar device can be successfully be used as a tool for personal diagnosis and the integration of sensoric components into these devices is a promising idea. Because BChE is a relevant marker, the demonstrated assay is expected to have a practical use.

## 2. Experimental Section

### 2.1. Plasma Samples

Plasma from inbred BALB/c mice was chosen for testing the method. In total 12 female mice were purchased from Velaz (Unetice, Czech Republic) and used for plasma preparation. The whole experiment was approved by the ethical committee of the Faculty of Military Health Sciences (University of Defence, Hradec Kralove, Czech Republic). On the day of collection, the mice weighed 19 ± 1 g and were two months old. For the whole time, the mice were kept in in a room with temperature 22 ± 2 °C, humidity 50% ± 10% and light/dark period each 12 h. The animals had no limitations in access to chow and water for the whole time. At the age of two months, the mice were narcotized by carbon dioxide and sacrificed by cutting of the jugular vein. The fresh blood was collected directly into tubes with lithium heparin (Dialab, Prague, Czech Republic). Plasma was acquired by centrifugation at 1500 RPM for 5 min. The fresh plasma samples were pooled together and stored at −80 °C until the assay.

### 2.2. Standard Assay of BChE Activity

Pure BChE (expressed in goat, received as lyophilized powder with enzyme activity ≥500 U/mg of protein) from Sigma-Aldrich (Saint Louis, MO, USA) diluted in pH 7.4 phosphate buffered saline (PBS, Litolab, Chudobin, Czech Republic) was used as a standard sample. The pooled plasma sample described above was used undiluted or diluted by PBS as well. The assay was performed in standard disposable PS cuvettes with optical length 1 cm. 5,5′-Dithiobis-(2-nitrobenzoic) acid (400 µL, 1 mmol/L), standard BChE enzyme solution or plasma sample (100 µL), PBS (pH 7.4, 400 µL) were added to the cuvette. After that, butyrylthiocholine chloride (100 µL, 50 mmol/L) was injected and absorbance at 412 nm was measured immediately and then after two minutes. The extinction coefficient ε = 14,150 L·mol^−1^·cm^−1^ for 5-thio-(2-nitrobenzoic) acid in pH 7.4 was used for enzyme activity calculation.

### 2.3. Assay Measured by Smartphone

**Scheme 1:**
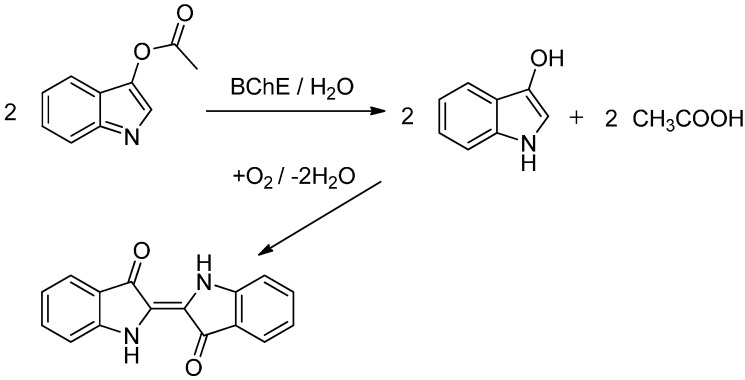
Principle of the indoxylacetate-based assay used for the determination of BChE activity.

Prior to the assay, strips were made by cutting filter papers 1PS (Whatman, Little Chalfont, UK). Indoxylacetate (Sigma Aldrich, St. Louis, MO, USA) was dissolved in pure ethanol up to a concentration of 10 mmol/L. The choice of paper and substrate was inspired by previous work where a cholinesterase-based dipstick was constructed and this type of paper and substrate was selected and successfully used [18]. The cut paper was immersed into the indoxylacetate solution for 5 min, and then it was pulled out and dried in the dark under standard ambient laboratory conditions (SATP). The prepared papers were kept in dark until use. Assayed plasma samples or BChE solutions (20 µL) were applied to the paper surface. The principle of the chromogenic reaction is described in [18,19]. In the reaction, indoxylacetate was hydrolyzed by cholinesterase and indigo blue was consequently formed (Scheme 1).

**Figure 1:**
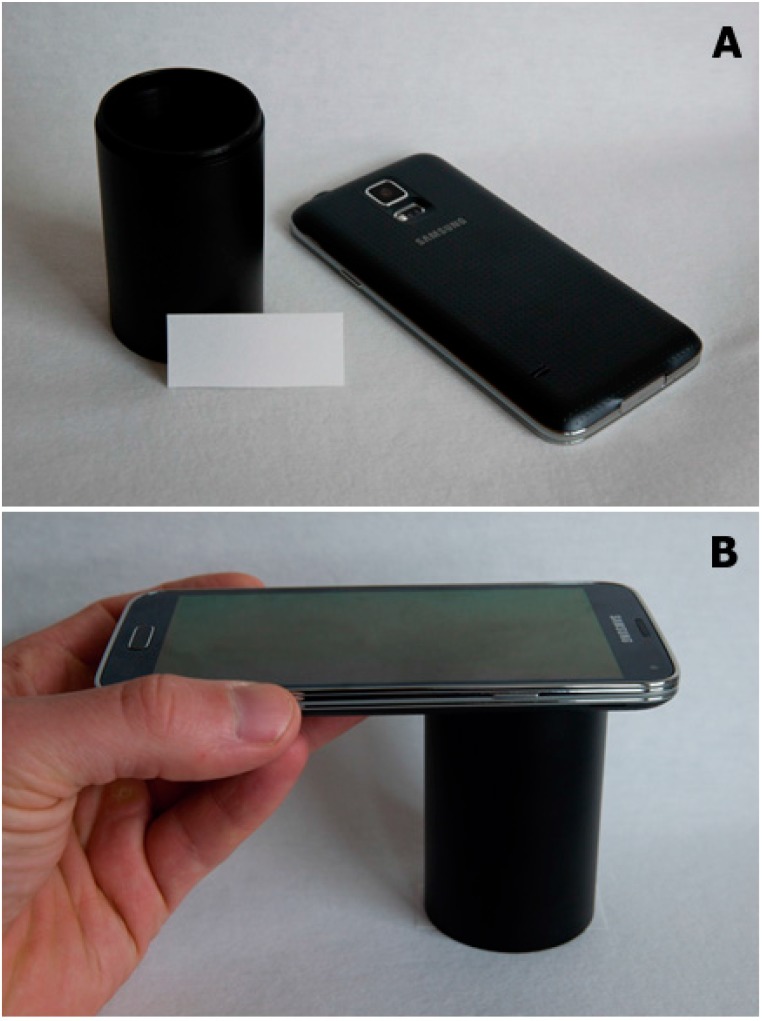
Photos of gear used for the assay. (**A**) Smartphone beside distance tube and paper soaked with indoxylacetate. The camera and LED light source are visible in the photo; (**B**) Setup used for photographing the paper.

The reaction was photographed using a Galaxy S5 smartphone (Samsung, Seoul, Korea). The automatic regime with forced flash (the LED light integrated into the smartphone) was chosen. The smartphone was placed on a black tube (seen in Figure 1). The second end of the tube was placed on the cut of paper and a collection of pictures was taken in regular intervals 0–5–10–15–20–25–30–35–40–45–50–55–60 min. The placement of the camera and paper prevented illumination by any other light source except the LED light. The setup of the device is depicted in Figure 1.

### 2.4. Data Processing

Photographs captured by the smartphone were processed using Zoner Photo Studio 17 (Brno; Czech Republic). Processed photographs were opened in the Editor Application of the software. The Color Shift function was chosen and RGB color code was read by clicking in half of a semi-diameter of the analyzed dots. GIMP 2.8.14 (a free open source software) was used as the second program for picture processing. RGB color code was obtained using the Color Picker function. The site for color reading was the same one used above.

The camera used took photographs in jpg format which has an 8-bit color scale (values of color tones 0–255, for a total of 256 color tone values; further indicated as color intensity) for each of the three (R, G, B) color channels. In an example, the R value equal to 0 is the darkest red, while the R value 255 is the brightest red. The principle of color use are described *e.g*., in work of Prats-Montalban and coworkers [20].

All measurements were made in pentaplicate (*n* = 5) and experimental data acquired by the aforementioned graphical software were further processed in Origin 9.1 (OriginLab Corporation, Northampton, MA, USA). Enzyme activity was expressed in katals where one katal respond to 1 mol of butyrylthiocholine converted by the BChE per 1 s. Color values were used either directly as obtained from the graphical software (test of time) or as a difference between the beginning of the experiment (*t* = 0 min) and time of sufficient coloration. Signal *vs.* noise equal to three criteria (*S/N* = 3) was considered for the limit of detection calculation. The ANOVA test was used for comparison between series of repeated measurements.

## 3. Results and Discussion

Pooled plasma samples were measured for BChE activity using the standard Ellman assay. Activity of BChE for butyrylthiocholine (final concentration in cuvette 5 mmol/L) was (2.73 ± 0.17) × 10^−5^ kat/mL of plasma. Two-fold serial dilutions of plasma were prepared (2.73 × 10^−5^ − 1.37 × 10^−5^ − 6.83×10^−6^ − 3.41 × 10^−6^ − 1.71 × 10^−6^ − 8.57 × 10^−7^ kat/mL) using PBS. The serial dilutions were used in subsequent experiments for calibration and validation purposes.

**Figure 2:**
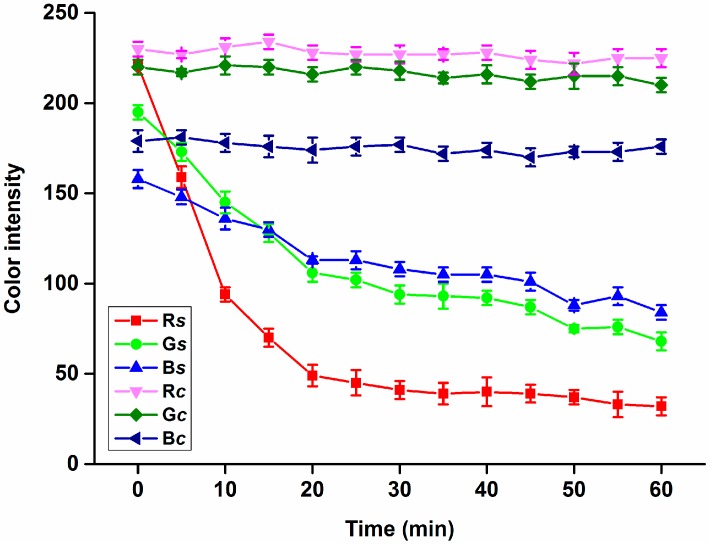
Performance of the smartphone for R, G and B channel color intensity change measurements with time. R*s*, G*s* and B*s* were acquired by assay of plasma samples on filter paper treated with indoxylacetate while R*c*, G*c* and B*c* were determined by assay of plasma samples on papers without any treatment. Error bars indicate standard deviations for *n* = 5.

In the first part of the assay based on a smartphone, coloration of the modified filter paper was measured in time interval 5 min from 0 to 60 min because of the undiluted plasma samples used. Results from the measurement are depicted as Figure 2. No significant (ANOVA test) change was observed when papers without indoxylacetate modification were used, *i.e.*, no change of color was observed in the control papers. Compared to the control papers, plasma applied on the surface of the papers treated with indoxylacetate caused an intensive coloration. The highest color value changes were perceived for the R channel, where the color value dropped from (average) 221 at the beginning to 41 after 30 min and finally 32 after 60 min. Changes for the G and B channels were much lower compared to the R oned. The color value for G dropped from 195 (0 min) to 68 (60 min) and the B value dropped from 158 (0 min) to 84 (60 min). Considering the results, the R channel seems to be the best one for scaling the assay based on indoxylacetate. Due to the time needed to run the reaction, an interval lasting 30 min seems to be suitable for BChE activity determination, as the color value change is minimal after this interval, so a 30 min interval was chosen for the subsequent experiments. Though choosing a longer interval would appear promising, the use of a long interval would be also disadvantageous because of sample desiccation. Low wettability of the paper used by aqueous solutions such as plasma samples and good wettability by ethanol solutions containing indoxylacetate was an advantage and reason why the drops with sample did not disappear because of absorption.

Small picture cuts containing 2 pixels (the minimal cutting size in Zoner Photo Studio 17) were prepared. Data were processed in Zoner Photo Studio 17 and GIMP 2.8.14 and color intensity values were determined. In compliance with expectation (color codes are universal for the same picture format), there was no difference between the color intensity values for the same pixels. From this point of view, both software products can be used with equal results.

**Figure 3:**
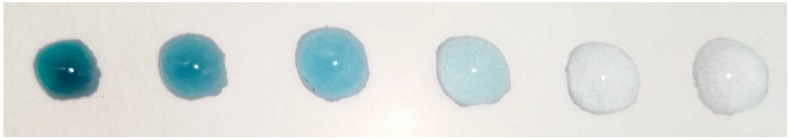
An example based on calibration using two-fold serial dilutions of plasma. Undiluted plasma is the left drop; the right drop is 32 times diluted plasma. The photograph was taken in the dark (in a black distance tube) using the smartphone chosen for the experiments and integrated LED flash as a sole source of light.

Calibration was performed using the aforementioned protocol and the chosen 30 min time interval. Two-fold serial dilutions of plasma (mentioned above) were used as a standard for calibration purposes. An example of the calibration is depicted in Figure 3.

**Figure 4:**
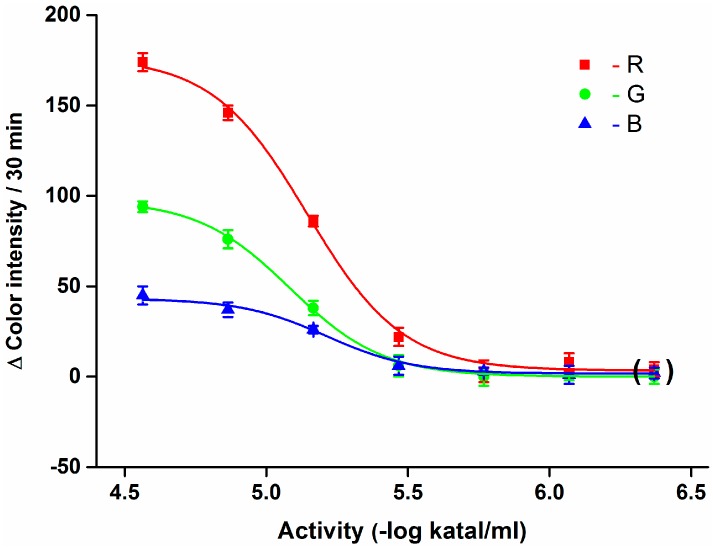
Calibration of the smartphone-based assay using diluted plasma samples. Differences of color intensity for the R, G and B channels are given. These were acquired by processing photographs taken at the beginning (0 min) of the assay and after 30 min. Points in brackets was taken by assay of pure PBS. Error bars indicate standard deviations for *n* = 5.

A quite intensive coloration is visible for undiluted plasma (activity 2.73 × 10^−5^ kat/mL) and plasma diluted two, four as well as eight times (activity 1.37 × 10^−5^, 6.83 × 10^−6^ and 3.41 × 10^−6^ kat/mL) also had perceptible coloration. Full calibration is shown in Figure 4. The limits of detection were not equal when the three color channels were compared. The lowest limit of detection—3.09 × 10^−6^ kat/mL—was achieved for the R channel. Analysis of the G and B channels provided worse limits of detection than analysis of the R channel, as limits of detection of 4.36 × 10^−6^ kat/mL for the G channel and 4.67 × 10^−6^ kat/mL for the B channel were found, that is, the limit of detection was approximately two to four times lower (depending on the right source) compared to visual detection. From this point of view, the camera-based assay is better than the assay by naked eye. On the other hand, the limit of detection is not the only advantage when the two approaches are considered. The camera-based assay has good repeatability and allows easy reproducibility, while subjective reading by the naked eye has significant disadvantages regarding the repeatability and reproductibility.

The coefficient of determination was equal to 0.996 for the R channel (Boltzmann function). The other channels had a close correlation as well. The G channel had a coefficient of determination of 0.998 and the B channel 0.989. Use of the R channel seems to be the best for the BChE activity assay and it was chosen for the subsequent experiments. The finding is quite surprising because the B channel would normally be expected to be the best when the visual coloration is considered. In this experiment, the B channel is preferred because of manual data processing; however, the other channels may be used for, *e.g*., control purposes in the future when the data processing is automated. When the data was compared to calibration using standard spectrophotometry and Ellman’s method, the limits of detection were slightly worse than for the spectrophotometry method where a limit of detection of 1.77 × 10^−6^ kat/mL was proved at the same time. It has to be emphasized that the limits of detection achieved are suitable to perform the assay in routine diagnosis because the limit of detection is several times below the expected BChE level in plasma because standard cholinesterasemia lays in a narrow range [10].

Besides the use of plasma, the method was tested for pure BChE in order to verify the correctness of the assay. Three mixtures of pure BChE were prepared and activity in the samples was adjusted to correspond to plasma dilutions of 2.73 × 10^−5^, 1.37 × 10^−5^, and 6.83 × 10^−6^ kat/mL. The differences (30 min interval) of color intensity for the R channel were 176 ± 7 for BChE 2.73 × 10^−5^ kat/mL, 144 ± 5 for BChE 1.37 × 10^−5^ kat/mL, and 84 ± 8 for BChE 6.83 × 10^−6^ kat/mL. The data differences between the plasma assay and the equivalent activity of BChE were insignificant. The result suggests that the smartphone-based assay is correct and no matrix effect occurs when biological samples are analyzed. Interference was tested as well. BChE was dissolved in PBS containing either 1 mmol/L of ascorbic acid, 1 mmol/L glutathione, 1 mmol/L of glucose, 1 mg/mL of bovine albumin, or 1 mg/mL of keyhole limpet hemocyanin. No differences in the assayed signal were seen when compared to the solution of BChE in PBS. The pure compounds (ascorbic acid, glutathione, glucose, albumin and hemocyanin) did not cause spontaneous coloration. Due to this finding, it can be stated that the method is not sensitive to interferences.

**Figure 5:**
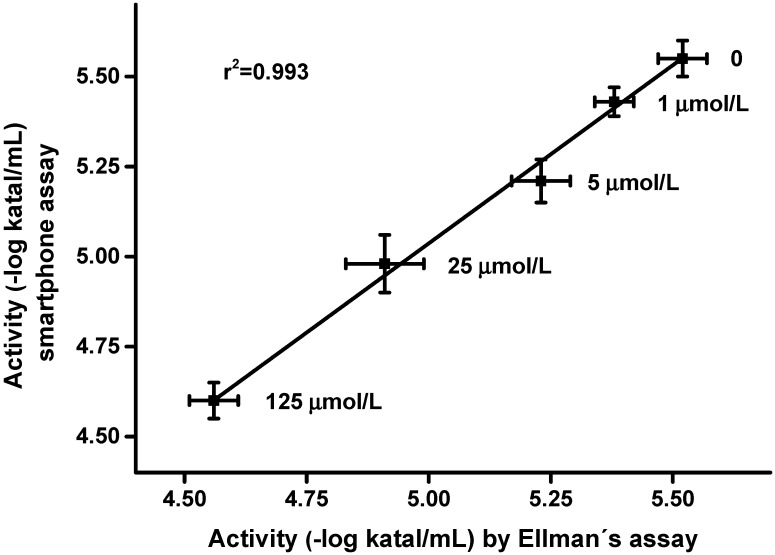
Validation of the smartphone-based assay using plasma samples spiked with carbofuran. Standard Ellman’s assay was used as a reference. The concentration of carbofuran in the plasma sample is written beside the experimental point. Error bars indicate standard deviations for *n* = 5.

Validation of the new method was made using standard plasma samples with addition of carbofuran, which is a pseudoirreversible inhibitor of the both AChE and BChE used as an insecticide in the past [21,22,23]. It was chosen as a representative insecticide for the purposes of this experiment. The validation is shown as Figure 5. In total 10 µL of carbofuran was injected per 990 µL plasma sample and the smartphone-based assay as well as Ellman’s assay were performed after 10 min of incubation. The two methods were mutually correlated and w quite high coefficient of determination of 0.993 was achieved.

The results confirm the applicability of the smartphone-based assay for a routine determination of cholinesterasemia in individual or field diagnoses. The limit of detection and total assay time are not better when compared to the standard Ellman’s assay, but the method is suitable for the determination of hypocholinesterasemia where approximately 30% and more decreases in BChE activity can be expected [10]. It should be emphasized that the total costs per assay are quite low because the cut filter papers and indoxylacetate needed per assay cost less than 1 Euro cent. In recent times, smartphones have been recognized as a promising tool for measurements based on image processing [24,25]. The current work confirmed the applicability of smartphones for a simple diagnosis with minimal costs per assay. The assay can be further easily improved and the limit of detection lowered by changing the output format of the photographs. Since the jpg format only provides 8-bits per channel, the colors are restricted to 256 values. Processing of raw photo format or tiff format would improve the number of assayed color values, e.g., 12-bit raw photo format or tiff format provide 4096 color values, and 16-bit 65,536 values. Unfortunately, most of current smartphones including the one used in the experiments offer 8 bit jpg as the only output format. Bigger camera sensors with a better dynamic range and better sources of light would be another improvement of the assay.

## 4. Conclusions/Outlook

Smartphones and similar devices equipped with integrated cameras and CPUs allowing data processing can be used as a simple tool in analytical chemistry. Their ready availability and low prices compared to more specialized analytical devices favor them to be used as an analytical or diagnostic tool under field or home conditions with quite good expected results. In agreement with the opinion of the author, a growing impact of analytical or diagnostic methods based on smartphones can be expected in the future.
